# Entropy Exchange and Thermodynamic Properties of the Single Ion Cooling Process

**DOI:** 10.3390/e21070650

**Published:** 2019-07-01

**Authors:** Jian-Guo Miao, Chun-Wang Wu, Wei Wu, Ping-Xing Chen

**Affiliations:** Interdisciplinary Center for Quantum Information, National University of Defense Technology, Changsha 410073, China

**Keywords:** thermodynamic properties, entropy exchange, laser cooling, single trapped ion

## Abstract

A complete quantum cooling cycle may be a useful platform for studying quantum thermodynamics just as the quantum heat engine does. Entropy change is an important feature which can help us to investigate the thermodynamic properties of the single ion cooling process. Here, we analyze the entropy change of the ion and laser field in the single ion cooling cycle by generalizing the idea in Reference (*Phys. Rev. Lett.* **2015**, *114*, 043002) to a single ion system. Thermodynamic properties of the single ion cooling process are discussed and it is shown that the Second and Third Laws of Thermodynamics are still strictly held in the quantum cooling process. Our results suggest that quantum cooling cycles are also candidates for the investigation on quantum thermodynamics besides quantum heat engines.

## 1. Introduction

Investigation into the thermodynamic properties of a quantum system has been a topic of interest in recent years [1,2,3,4,5,6,7,8,9,10]. To expand on this topic, a suitable platform is needed. In classical thermodynamics, a typical heat engine that contains a complete thermodynamic cycle plays an important role in theoretical investigations of thermodynamic devices. Correspondingly, in the quantum regime, a real quantum heat engine may be a suitable platform for the study of quantum thermodynamics [11,12,13,14,15,16,17]. However, how to set up a real quantum heat engine whose working agent is made up of several atoms is still under study and only a few mature examples have been realized in experiments [11].

In the field of classical thermodynamics, the refrigerator is also a typical model. As the inverse process of the heat engine, the laser cooling process can also serve as a suitable platform for discussing the thermodynamic properties of the quantum system. After the birth of laser in the 1960s, a number of laser cooling schemes were proposed by physicists, such as Doppler cooling [18,19], polarization gradient cooling [20], Sisyphus cooling [21], magnetically induced laser cooling [22], sideband cooling [23,24], electromagnetically induced transparency [25], bichromatic force cooling [26] and so forth. Among these cooling schemes, the cooling of a single ion is also involved, which can serve as a suitable platform for exploring the thermodynamic properties of the quantum system. It is well known that both the motional energy and the entropy of the atom can be transported into the environment during the atom cooling process. As an important thermodynamic parameter of the system, entropy characterizes a system’s capability to dispose information and the entropy change of the system features the degree of irreversibility of the evolution. So, the discussion on the entropy exchange can be a suitable cut-in point to investigate the thermodynamic features of the ion cooling cycle.

**Figure 1:**
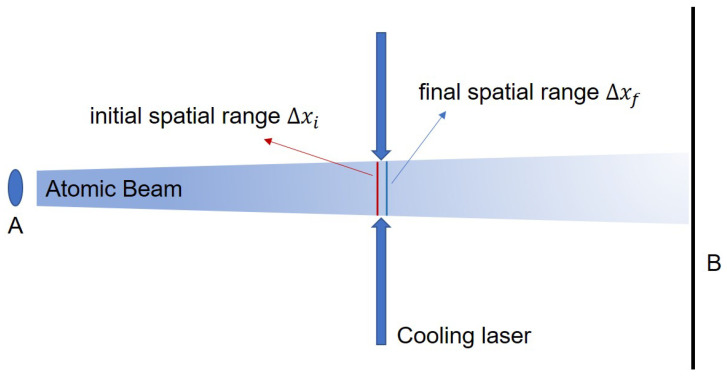
(color online). The sketch map of the laser cooling scheme described in Reference [28]. The atomic beam is launched from the atom source (**A**) with an initial transverse velocity range $\Delta v_{i}$. The transverse velocity range of the atomic beam is compressed by the cooling laser, which is orthogonal to the atomic beam, while the spatial range almost remains unchanged $\Delta x_{i}\approx \Delta x_{f}$. The transverse velocity range can be obtained by analysing the images recorded by the beam imaging detector (**B**).

To discuss the entropy exchange between different parts of the cooling cycle, we should choose a suitable scheme to calculate the entropy of the system. In 2008, Harold Metcalf proposed a new method for calculating the entropy exchange during the bichromatic force cooling process and put forward a new viewpoint that the stimulated emission can also take the responsibility of cooling atoms and the laser can absorb the entropy lost by the atomic system [27]. Then in 2015, the atomic beam collimation experiment [28] provided powerful evidence to confirm his viewpoint (shown in Figure 1).

The calculation scheme is based on the bichromatic force cooling scheme [26,29] which contains no spontaneous emission. The system is divided into two main parts, the atomic ensemble and the laser field. Based on the definition of classical entropy, the entropy change of the atomic ensemble is represented by the reduction of the phase space,

(1) $$\Delta S_{a}=k_{B}\ln\frac{\Delta v_{f}\cdot \Delta x_{f}}{\Delta v_{i}\cdot \Delta x_{i}},$$

where $k_{B}$, $\Delta v_{i}$ $\left(\Delta v_{f}\right)$ and $\Delta x_{i}$ $\left(\Delta x_{f}\right)$ denote Boltzmann constant, the velocity range and spatial range of the atomic ensemble before (after) the cooling process. After the interaction, the velocity range of the atoms decreases while the spatial scope remains almost unchanged, so it is clear that the entropy of the atomic ensemble reduces, $\Delta S_{a}<0$ [27,28]. For the laser field, assume that its initial state is described by a coherent state $\rho_{l}^{i}=\left|\alpha \rangle \langle \alpha \right|$, so its initial entropy is zero, remembered as $S_{l}^{i}=0$. As there is no spontaneous emission in the bichromatic force cooling scheme, the final state of the laser is a mixed state of a set of coherent states [27]. The maximum photon number needed to cool the atom is denoted by $\Delta n=Mv_{c}/\hbar k$, where *M* is the mass of the atom, *k* the wave vector of laser, $v_{c}$ the capture speed of the cooling scheme. Thus the average photon number of the laser field is between $n={\left|\alpha \right|}^{2}$ and $\left(n+\Delta n\right)$, in other words, the mixed state consists of a set of states whose average photon number is bigger than *n* and smaller than $\left(n+\Delta n\right)$ with equal probability. To distinguish two adjacent states |α〉, |β〉, the overlap of them should satisfy the condition ${\left|\langle \alpha |\beta \rangle \right|}^{2}=e^{{-|\alpha -\beta |}^{2}}\leq e^{-1}$, which means that the average photon number of them should differ at least by $\pm 2\sqrt{n}$, where $n={\left|\alpha \right|}^{2}$. Thus, we get the number of the possible states of laser $N=\Delta n/2\sqrt{n}$ and the final entropy of the laser,

(2) $$S_{l}^{f}=k_{B}\ln N\;.$$

As the initial entropy of the laser is zero, we have $\Delta S_{l}=S_{l}^{f}=k_{B}\ln N$. It has been proved that the entire entropy change, $\Delta S=\Delta S_{atom}+\Delta S_{l}>0$, roughly satisfies the Second Law of Thermodynamics [27,28].

The calculation scheme described above provides a feasible method for quantitatively analyzing the entropy exchange between the atomic ensemble and the laser field, which would be helpful for investigating the thermodynamic properties of quantum systems. But this calculation scheme is proposed for the atomic ensemble whose spatial range and velocity range can be obtained intuitively just as depicted in Figure 1. For the quantum system containing only one single ion, it is hard to measure or calculate its spatial and velocity ranges. So, it is necessary to propose a new entropy calculation scheme.

In this paper, we generalize the calculation scheme proposed in Reference [27] to the single ion cooling process. Based on the entropy calculation scheme, we analyze the entropy exchange between the single trapped ion system and the laser field, and confirm that the laser cooling process satisfies the Second and Third Laws of Thermodynamics strictly.

## 2. Calculation and Results

**Figure 2:**
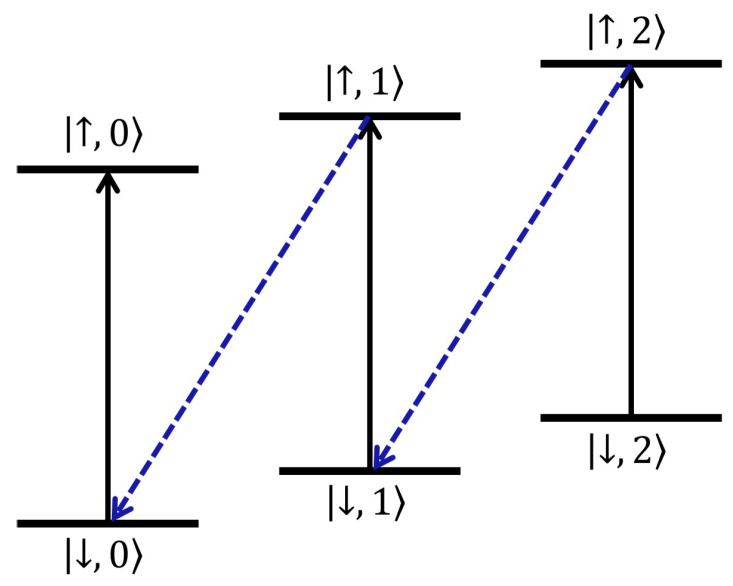
(color online). The sketch map of a complete cooling cycle. Interacting with the bichromatic laser field, the ion in non-zero phonon states will lose a phonon, while the ion initialized in vacuum state will keep unchanged in finite time. Finally, the ion in spin-up state |↑,0〉 would return to the spin-down state |↓,0〉.

Here, we consider a cooling cycle for a single trapped ion system. The single trapped ion is confined in a linear Paul trap and its internal states are denoted by the spin-like states |↓〉 and |↑〉 (mostly a metastable state). The specific process of the cooling cycle is described as follows (see Figure 2).

**(i)** First, the internal state of the ion is initialized at ground state |↓〉 while the external state is prepared in a thermal state $\rho_{ion}^{i}=\sum_{n=0}^{\infty }P_{n}\left|n\rangle \langle n\right|$ which satisfies the thermal distribution [30],

(3) $$P_{n}=\frac{1}{\bar{n}+1}\cdot {\left(\frac{\bar{n}}{1+\bar{n}}\right)}^{n},\;n=0,1,2,\cdots .$$

**(ii)** Then implement a π pulse carrier transition followed by a π pulse blue-detuned transition. It is generally known that the Hamiltonian of blue-sideband transition is in compliance with the anti-Jaynes-Cummings model, and the Rabi frequencies of different transitions between |↓,n〉 and |↑,n+1〉 are dependent on the phonon number *n* as the form $\sqrt{n+1}\eta \Omega$, where η is the Lamb-Dicke parameter, Ω the Rabi frequency of carrier transition. To ensure the entire process is coherently manageable, we should erase the influence of the phonon number. By taking the advantage of the adiabatic passage method and transitionless quantum driving scheme [31,32,33,34,35], a fast and complete blue-sideband transition can be achieved independently of the initial phonon state. The detailed conditional settings are described in Reference [32] and a complete cooling cycle is also known as a subtraction operation. After the interaction, the ion in state |↓,n〉 is transformed into |↓,n−1〉(n≥1), while the ion initialized in |↓,0〉 would stay in the spin-up state |↑,0〉.

Furthermore, for the blue-detuned laser field, a coherent state $\rho_{laser}^{i}=\left|\alpha \rangle \langle \alpha \right|$ can be the closest description. So we assume that the laser field is prepared in a pure state whose entropy is zero. The laser field would interact with the non vacuum states |↓,n〉(n≥1) and be transformed into a new state called a single-photon added state, denoted as |α,1〉. As the ion is probabilistically in the vacuum state, the final state of the laser field is a mixed state of |α〉 and |α,1〉 [36]. The density matrix of the entire system would evolute as below,

(4) $$\rho^{i}=\sum_{n=0}^{\infty }P_{n}\left|↓,n\rangle \langle ↓,n\right|⊗\left|\alpha \rangle \langle \alpha \right|\Rightarrow \sum_{n=1}^{\infty }P_{n}|↓,n-1\rangle \langle ↓,n-1|⊗|\alpha ,1\rangle \langle \alpha ,1|+P_{0}\left|↑,0\rangle \langle ↑,0\right|⊗|\alpha \rangle \langle \alpha |.$$

**(iii)** Finally, the ion in |↑,0〉 would fall into the stable state |↓,0〉 through spontaneous emission or some other partially reversible process. Then there comes the end of a single cooling cycle, and we have the final density matrix of the entire system,

(5) $$\rho^{f}=P_{0}\left|↓,0\rangle \langle ↓,0\right|⊗\left|\alpha \rangle \langle \alpha \right|+\sum_{n=1}^{\infty }P_{n}|↓,n-1\rangle \langle ↓,n-1|⊗|\beta \rangle \langle \beta |.$$

So far, we have exhibited a complete cooling cycle of a single trapped ion. Based on this process, we investigated the thermodynamic properties of the quantum system by calculating its entropy change. In the following discussion, we will focus on the entropy change of the single ion and the blue-detuned laser field. Partial tracing the final density matrix $\rho^{f}$, we have the final external state of the ion:

(6) $$\rho_{ion}^{f}=\left(P_{0}+P_{1}\right)\left|0\rangle \langle 0\right|+\sum_{n=1}^{\infty }P_{n+1}|n\rangle \langle n|,$$

and the density matrix of the laser field:

(7) $$\rho_{laser}^{f}=P_{0}\left|\alpha \rangle \langle \alpha \right|+\left(1-P_{0}\right)|\alpha ,1\rangle \langle \alpha ,1|,$$

The single-photon-added coherent state (SPACS) |α,1〉 is the result of a successive elementary one-photon excitation of a classical coherent field [36], denoted as

(8) $$|\alpha ,1\rangle =\frac{{\hat{a}}^{†}|\alpha \rangle }{\sqrt{1+{\left|\alpha \right|}^{2}}}.$$

Similar to most laser cooling schemes, the ion is cooled after the implementation of the cooling cycle and its entropy also decreases. In this article, we adopt von Neumann entropy to evaluate the entropy of the system which can be a thermal state or a non-thermal state. Combining the definition of von Neumann entropy [37] with the above analysis, we can simply calculate the entropy change of the ion subsystem. The initial and final states of the ion are described by Equations (3) and (6), so we have the initial and final entropy of the ion subsystem, respectively:

(9) $$S_{ion}^{i}=-k_{B}\sum_{n=0}^{\infty }P_{n}\ln\left(P_{n}\right),$$

(10) $$S_{ion}^{f}=-k_{B}\left[\left(P_{0}\;+\;P_{1}\;\right)\ln\left(P_{0}+P_{1}\right)+\sum_{n=2}^{\infty }P_{n}\ln\left(P_{n}\right)\right].$$

The entropy of the ion reduces as,

(11) $$\begin{array}{cc} \Delta S_{ion} & =S_{ion}^{f}-S_{ion}^{i}\\ & =-k_{B}\left[\left(P_{0}+P_{1}\right)\ln\left(P_{0}+P_{1}\right)-P_{0}\ln\left(P_{0}\right)-P_{1}\ln\left(P_{1}\right)\right]. \end{array}$$

The initial laser field is denoted by a coherent state, and its entropy is zero,

(12) $$S_{laser}^{i}=0.$$

After interacting with the ion, the laser filed becomes a mixed state of |α〉 and |α,1〉, which is represented by Equation (7). To calculate the final entropy of the laser field, we should confirm whether these two states |α〉 and |α,1〉 are nearly orthogonal to each other. To estimate the orthogonality of these two states, we compute the overlap of them,

(13) $${\left|\langle \alpha |\alpha ,1\rangle \right|}^{2}={\left|\langle \alpha |\frac{{\hat{a}}^{†}}{\sqrt{1+{\left|\alpha \right|}^{2}}}|\alpha \rangle \right|}^{2}=\frac{{\left|\alpha \right|}^{2}}{1+{\left|\alpha \right|}^{2}}.$$

Choose the “distinct state” criterion noted in Reference [27], which asks the overlap formula ${\left|\alpha \right|}^{2}{/(1+|\alpha |}^{2})\leq e^{-1}$. Then, this criterion requires the effective average photon number of the laser field to have the value $m\equiv {\left|\alpha \right|}^{2}\leq \frac{1}{e-1}$.

As described, the distinguishability of |α〉 and |α,1〉 is related to the average photon number of the relevant laser field. In most cases, the ion is located in the laser spot but not all photons interact with ions. So it may be unfair to involve the whole laser field in consideration. Characterizing the interaction as the collisions between photons and the single trapped ion, the relevant laser field would be confined in a cylinder whose cross-section and length are denoted as σ and $ct_{cool}$, where σ is the off resonance absorption cross-section [38], $t_{cool}$ the duration of the cooling cycle. As the interaction is blue-detuned, σ is significantly smaller than the size of the atom $\sigma \ll \pi r^{2}$, where *r* is the atomic radius.

For a beam of intensity *I*, we find $I=m\hbar \omega /\sigma ct_{cool}$, thus have the average photon number of the relevant laser field satisfies: $m=I\cdot \sigma ct_{cool}/\hbar \omega$. It can be proved that the laser intensity of most experimental setting of the cooling process [32] satisfies the condition: $m\leq \frac{1}{e-1}$. So we can confirm that states |α〉 and |α,1〉 are distinguishable. We can calculate the final entropy of the laser field according to Equation (7),

(14) $$S_{laser}^{f}=-k_{B}\left[P_{0}\ln\left(P_{0}\right)+\left(1-P_{0}\right)\ln\left(1-P_{0}\right)\right].$$

Thus we have the entropy exchange of the laser field

(15) $$\begin{array}{cc} \Delta S_{laser} & =S_{laser}^{f}-S_{laser}^{i}\\ & =-k_{B}\left[P_{0}\ln\left(P_{0}\right)+\left(1-P_{0}\right)\ln\left(1-P_{0}\right)\right]. \end{array}$$

Until now, we have obtained the entropy change of the ion and the laser field in a complete cooling cycle. Then, combining Equations (11) and (15), the entropy change of the whole system can be described as follows,

(16) $$\Delta S=\Delta S_{ion}+\Delta S_{laser}+S^{\prime },$$

where $S^{\prime }$ is the entropy production of the process |↑,0〉→|↓,0〉. If we can find a reversible method to carry out the transition, $S^{\prime }=0$, otherwise, $S^{\prime }>0$. In order to simplify the question, we assume that there exists a reversible operation converting the spin-up state to spin-down state, thus we can ignore the effect of $S^{\prime }$ and Equation (16) can be written as,

(17) $$\Delta S=\Delta S_{ion}+\Delta S_{laser}\;.$$

Combining Equations (3), (11), (15) and (16), it is clear that ΔS is related to the initial average phonon number $\bar{n}$.

**Figure 3:**
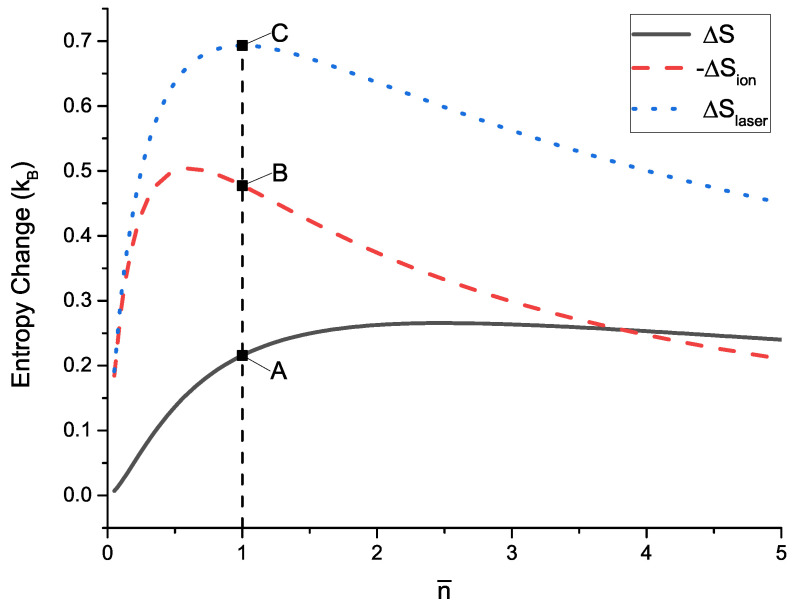
(color online). Entropy change of the system after a complete cooling cycle with different initial average phonon number. Entropy increment of the entire system: ΔS (black solid line); entropy reduction of the ion: $-\Delta S_{ion}$ (red dashed line); entropy increment of the laser field: $\Delta S_{laser}$ (blue dotted line). For example, if the initial average phonon number is $\bar{n}=1$, after a cooling cycle, ΔS, $-\Delta S_{ion}$, $\Delta S_{laser}$ will be expressed by points A, B, and C, respectively.

For a single cooling cycle, the relation between $\bar{n}$ and ΔS is shown in Figure 3. It is clear that the laser field has the capacity to absorb the entropy lost by the ion during the cooling process which is in coincidence with the viewpoint in Reference [27]. The entropy of the entire system increases after the cycle even if $S^{\prime }=0$, which means the cooling cycle satisfies the Second Law of Thermodynamics. Moreover, from Figure 3, we can find that when the average phonon number approaches zero $\bar{n}\to 0$, the entropy lost by the ion subsystem tends rapidly to zero, which means it becomes more and more difficult to cool the ion further. So, the result here confirms that the single trapped ion can not be cooled to absolute zero within finite steps. In other words, the calculation result also shows that the Third Law of Thermodynamics is held in the quantum field.

## 3. Conclusions and Discussions

For the exploration of quantum thermodynamics, a complete cooling cycle of a single ion can also be a promising model just like a quantum heat engine is. We have performed a detailed calculation of the entropy change of a single trapped ion cooling process. Through analysis, we confirm the concept of “entropy capacity of laser” and show that the Second and Third Laws of Thermodynamics are well fulfilled in the laser cooling process of a quantum system. Our idea and method can be applied to analyze other cooling processes of the single ion system. In future research, we would like to define the efficiency of a cooling cycle from the perspective of entropy and consider the factors that would affect the cooling efficiency.
